# Development and use of a genitourinary pathology digital teaching set for trainee education

**DOI:** 10.4103/2153-3539.63822

**Published:** 2010-05-26

**Authors:** Li Li, Bryan J. Dangott, Anil V. Parwani

**Affiliations:** 1 Department of Pathology, Albany Medical Center, 43 New Scotland AveAlbany, NY, 12208, USA; 2 Center for Pathology Informatics, Department of Pathology, University of Pittsburgh Medical Center, Pittsburgh, PA 15232, USA

**Keywords:** Digital teaching set, pathology education, whole slide imaging

## Abstract

**Background:**

Automated, high-speed, high-resolution whole slide imaging (WSI) robots are becoming increasingly robust and capable. This technology has started to have a significant impact on pathology practice in various aspects including resident education. To be sufficient and adequate, training in pathology requires gaining broad exposure to various diagnostic patterns through teaching sets, which are traditionally composed of glass slides.

**Methods:**

A teaching set of over 295 glass slides has been used for resident training at the Division of Genitourinary Pathology, Department of Pathology, University of Pittsburgh Medical Center. Whole slide images were prepared from these slides using an Aperio ScanScope CS scanner. These images and case-related information were uploaded on a web-based digital teaching model.

**Results:**

The web site is available at: https://www.secure.opi.upmc.edu/genitourinary/index.cfm. Once logged in, users can view the list of cases, or search cases with or without diagnoses shown. Each case can be accessed through an option button, where the clinical history, gross findings are initially shown. Whole slide images can be accessed through the links on the page, which allows users to make diagnoses on their own. More information including final diagnosis will display when the diagnosis-button is clicked.

**Conclusion:**

The web-based digital study set provides additional educational benefits to using glass slides. Residents or other users can remotely access whole slide images and related information at their convenience. Searching and sorting functions and self-testing mode allow a more targeted study. It would also prepare residents with competence to work with whole slide images. Further, the model can be expanded to include pre-rotation and post-rotation exams, and/or a virtual rotation system, which may potentially make standardization of pathology resident training possible in the future.

## INTRODUCTION

Automated, high-speed, high-resolution whole slide imaging (WSI) robots are becoming increasingly robust and capable.[1] A number of commercial products provide affordable rapid image captures, supporting image viewer software and services.[2] This technology has started to have a significant impact on pathology practice in various aspects including primary histologic diagnosis,[3] primary frozen section diagnosis via telepathology,[4] teleconsultation,[5] pathology quality assurance,[6,7] pathology education,[8] and competency assessment of residents.[9]

Surgical pathology includes a variety of disease entities, each of which may microscopically present as various patterns and changes. Pathology residents are exposed mostly to the common or typical presentations. To be sufficient and adequate, training in pathology requires gaining broad exposure to these diagnostic patterns through teaching sets, which traditionally are composed of glass slides. Although pathology resident education benefits to a great extent from teaching sets of glass slides, the disadvantages of using glass slides are obvious, for example, colors of stains fade over time, glass slides can be easily broken or lost, the slides can be used only by one person at a time, and a microscope is needed.

A previous study has shown that whole slide images are sufficient for pathologists to make reliable diagnostic decisions.[1] The digital image quality and scan speed to acquire a whole slide image have improved since then. Therefore, whole slide imaging could provide additional educational benefits to using glass slides. We developed a web-based genitourinary (GU) pathology digital teaching set and evaluated its use for trainee education.

## METHOD

A teaching set of over 295 glass slides has been used for resident training at the Division of Genitourinary Pathology, Department of Pathology, University of Pittsburgh Medical Center (UPMC). The slides are kept at the division secretary's desk, and are singed out when a trainee rotates through the division. An accompanying word sheet with case numbers and diagnoses only are available as a hard copy or an electronic file. Further information, such as patient's age, clinical presentation, gross pathology, microscopic, and immunohistochemistry findings, can be manually retrieved from the Anatomic Pathology Lab Information System (APLIS) by the trainee.

Whole slide images were prepared using an Aperio ScanScope CS scanner (Aperio, Vista, CA) from these slides. A 20× scanning magnification was selected. During the scanning process, the slides were de-identified. The average scanning time per slide was 3-5 min depending on the size of the tissue area on the slide. The sizes of image files vary from 200 megabytes to 1 gigabyte. The whole slide images were uploaded to an image server, which uses a flash-based application. A web-based digital teaching model has been implemented at our institute using Oracle 11g (Oracle, Redwood Shores, CA) as the database server, SunOne (Oracle, Redwood Shores, CA) as the web server, ColdFusion (Adobe systems, San Jose, CA) as the programming language, and a web middleware program to dynamically display information from a database. The hardware where the servers reside has a standard server setup with a Qual Core Intel processor, 4 GB of Random access memory (RAM), and 10 TB of storage capacity. The system infrastructure and soft ware resources, provided by the Center for Pathology Informatics at UPMC, have been built and maintained by the center's Information Technology (IT) group. The optimal bandwidth for the image server to run is 100 mb, but it can be 10 mb.

Two web applications were built using this model. One is a web-based data entry tool (https://www.secure.opi.upmc.edu/genitourinary/generate2/logout.cfm). Privileges of accessing this site were given by the system administrator. Case-related information, including the patient's age and sex, brief clinical history, important gross and microscopic findings, ancillary studies, diagnosis and discussion, was retrieved from APLIS. Subsequently, the retrieved information and the links of corresponding whole slide images were entered into the system through the data entry tool. Additional functions are also available on this site, including modification of a study set, as well as generation and modification of pre-test and post-test questions and answers. The other web application is the end-user site, which was designed to allow the user to search cases based on a diagnosis or ICD-O site (International Classification of Diseases for Oncology, http://www.who.int/classifications/icd/adaptations/oncology/en/) to view or hide the diagnosis, and to access whole slide images via web links. With administrative support for the hardware, operating system, image, database and web servers provided by The Center for Pathology Informatics UPMC, this project was accomplished in 1 month by a group consisting of a pathology-informatics visiting resident, a pathology-informatics fellow, an attending pathologist and a programmer.

## RESULTS

**Figure 1:**
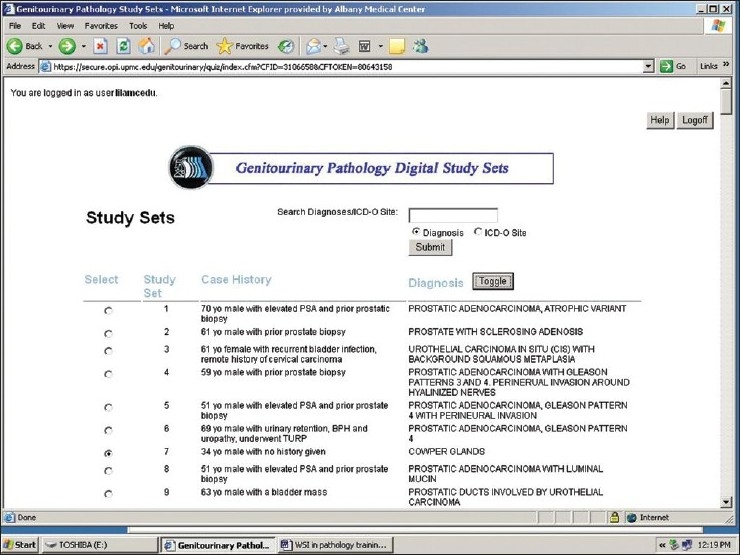
The list of cases is shown. The search function allows searching by diagnosis or ICD-O site. The diagnoses can be shown or hidden

**Figure 2:**
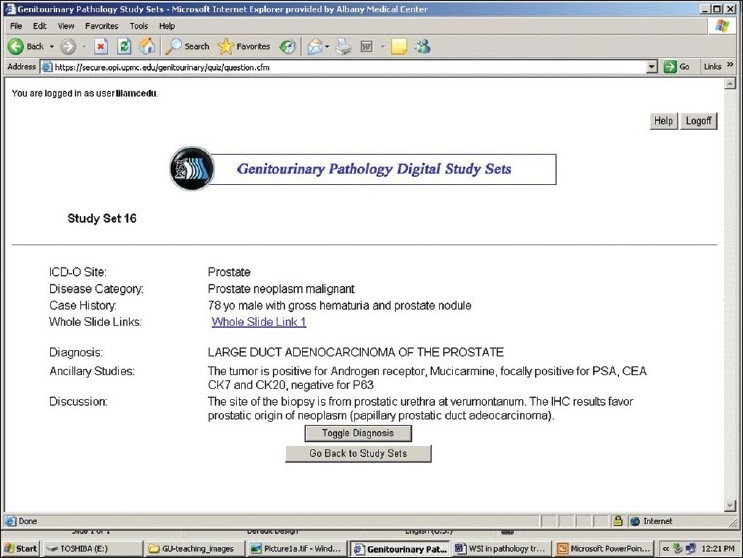
Once an option button is clicked, the ICD-O site, disease category, clinical history, gross findings and the link to whole slide images are shown. More information including microscopic findings, ancillary study result, final diagnosis, and discussion will display when the diagnosis-button is clicked

**Figure 3:**
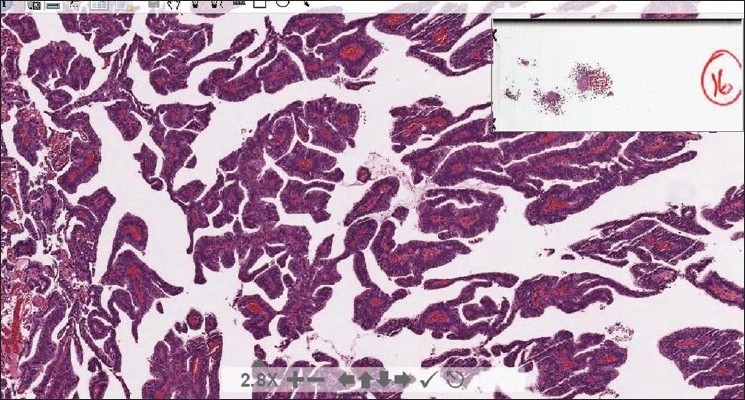
A whole slide image is displayed via spectrum webscope in a pop-up window. A tool bar (shown at the bottom of the screen) can be used to change the power and to move the display field

The web site is available at: https://www.secure.opi.upmc.edu/genitourinary/index.cfm. It requires registration and logging in. Once logged in, users can view the list of cases, and choose to show or hide the diagnoses. The search function allows searching by diagnosis or ICD-O site. A text box is associated with searching for diagnosis, whereas a drop-down list of ICD-O sites permits searching via these codes. An option button is associated with each case [Figure 1]. Clicking on an option button leads the display to the next page where the ICD-O site, disease category, clinical history and gross description (if available) are initially shown. Whole slide images can be accessed through the links on the page [Figure 2], which allows users to make diagnoses on their own. More information including microscopic findings, ancillary study results, final diagnosis, and discussion will display when the diagnosis-button is clicked. A whole slide image is displayed via Spectrum webscope (Aperio, Vista, CA) in a pop-up window when the image link is clicked. A tool bar can be used to change the power and to move the display field. Magnification can be increased by means of a double mouse click on the image [Figure 3].

## DISCUSSION

Whole slide imaging is a specific digital imaging modality that uses computerized technology to scan and convert pathology slides into digital images that can be viewed on a computer using viewing software. Viewing the digital images mimics a light microscopy, which allows a user to move (scan) from field to field and increase or decrease (zoom in/out) the magnification. Therefore, this is also known as “virtual microscopy”. In order to make whole slide images available online, one would also need to implement a web server and an image sever.

Since the entry of virtual microscopy into medical student education in 2000, there has been a progressive increase in the use of this technology in medical schools in the United States, as well as in international medical schools. One example of such applications is the virtual slide box (http://www.path.uiova.edu/virtualslidebox) developed at the Carver College of Medicine, University of Iowa, which is internally used for pathology teaching to small groups of medical students, and is also shared among institutions.[8] Several whole slide imaging-based applications have been implemented for the purpose of pathology education that can be useful in broader areas, including resident training. Virtual microscopy has been used in didactic live-audience lectures. Seamless transition between a power point presentation and virtual microscopy was provided by hyperlinking power-point slides to the virtual slide viewer.[10] Two web-based virtual microscopy programs, a digital atlas of breast histopathology and an interactive Gleason grading tool of prostate biopsies, have been developed at Tampere University in Finland.[11,12] Recently, a performance-based virtual slide competency examination has been developed at the Carver College of Medicine, The University of Iowa to measure pathology residents' morphologic diagnostic skill. Evaluation of this program showed that it would be a valid measure of an individual resident's progress in morphologic competency.[9]

Whole slide imaging technology and computer accessibility have advanced to the point that virtual microscopy can be integrated into a pathology residents' educational activities. The digital teaching set we developed provided additional benefits of using the glass slides. The whole slide images are stored in a central server repository, which would not be broken or lost with proper system management, such as periodic backup. The stain colors would not fade over time. The virtual slides can be remotely accessed at the user's convenience, and can be shared by multiple users concurrently. Searching and sorting functions are built in to allow more targeted study. The program can be used in a self-test mode or as a supplement to reading material. Constantly available case-related information eliminates the step of manual retrieval of information from a separate APLIS. The digital images can be annotated and the annotation can be displayed or hidden. All the slides are de-identified which makes sharing virtual slides among institutions possible.

The drawback of using virtual slides is that viewing virtual slides usually takes a longer period of time than viewing glass slides. The speed of loading digital images also is dependent on the speed of the user's network and computer. Since it is unlikely that virtual microscopy will replace glass slides entirely in the near future, pathology training should still focus on gaining capability of making effective diagnosis on glass slides. However, the American Board of Pathology has been using virtual slides for a subset of microscopic questions for a number of years.[13] In addition, virtual microscopy has been widely used in pathology practice as indicated earlier. Both suggest that a pathology resident should develop the capability of efficiently viewing virtual slides. Therefore, whole slide imaging should be a reliable and necessary component of pathology resident training. This pilot implementation demonstrates that a digital teaching set not only can provide rich teaching material to residents in a flexible, efficient, and reliable way, but also can prepare residents with the competence to work with whole slide images.

Further, the program can be expanded to include more components. The Accreditation Council for Graduate Medical Examination (ACGME) requires residency programs to implement objectively measurable performance-based education and assessment. It is critical for residents to develop skills in performing microscopic examinations to make accurate diagnosis during residency training. We plan to add a pre-test as a self-assessment tool and post-test as a measurement of residents' diagnostic competency. The pre and post-test entry tool has already been built in the data-entry application. The IT staff at the Center for Pathology Informatics UPMC has been continuously working to include the test module on the end user's site. The pathology team including residents, fellows, and pathologists will write up questions and answers according to the educational objectives. The program will also be built so that the performance of each trainee can be recorded and statistics can be done.

A virtual rotation of pathology informatics has been implemented at UPMC and the work was published in 2009.[14] The course includes didactic lectures given by experts in the fields and video-recorded hands-on laboratories. It can accommodate various rotation structures as a self-paced rotation and is available to all pathology residency programs. Some residency programs have started to incorporate this virtual rotation in their resident training. The same resource and expertise can be used to add didactic lectures to the digital teaching set program to create a virtual subspecialty rotation in anatomic pathology. Meanwhile, the UPMC department of pathology is an academic center with various subspecialties of surgical pathology. Its tremendous teaching material includes glass teaching sets of other subspecialties similar to the GU teaching set. Therefore, similar digital teaching sets can be developed based on this program model.

Last, it is well known that different pathology residency programs can have a different variety of specimens. Pathology residents may thus have uneven exposure to various types of specimens. With glass teaching slides, it is difficult to share teaching material among institutions. The digital teaching set we developed can be accessed remotely from anywhere with network connections. The step of de-identification is critical to make the whole slide images available to outside users. The digital teaching set along with its other possible components like pre and post-rotation exams and virtual rotation can provide valuable educational opportunities to pathology residents nationwide, which may potentially make standardization of pathology resident teaching possible in the future.
